# Why did not all studies conducted during Darfur’s armed conflict obtain ethics approval? Insights from a qualitative study

**DOI:** 10.1186/s12910-025-01194-5

**Published:** 2025-05-06

**Authors:** Ghaiath Hussein, Khalifa Elmusharaf

**Affiliations:** 1 School of Medicine, Trinity College Dublin, Dublin, Ireland; 2 Applied Health Research Institute, University of Birmingham Dubai, Dubai, United Arab Emirates

**Keywords:** Humanitarian ethics, Research ethics, Ethics review, Informed consent, Focus groups, Darfur, Sudan, United nations

## Abstract

**Background:**

Armed conflicts are associated with multiple factors that may deem applying the ethical standards of research conducted in war-affected areas hard to achieve, compared to research conducted in peace time.

**Objective:**

Using the example of studies conducted by the humanitarian agencies in the war-troubled region of Darfur, west Sudan between 2004 and 2012, a qualitative study was pursued to have a deeper understanding of the factors that affected the reporting of gaining the ethical approval in the published reports of these studies.

**Methods:**

A qualitative study was used that involved conducting interviews and focus groups with the relevant stakeholders, namely the representatives of the national and international non-governmental organizations, UN agencies, and the national humanitarian and research governance bodies in Sudan.

**Results:**

38 participants were involved (5 interviewees and 33 participants in the focus groups). The participants expressed a consensus on the need for an ethical oversight for research in the humanitarian settings in Sudan and particularly Darfur. Following a thematic analysis, four main themes were identified to explain why the humanitarian studies in Darfur were not submitted to formal ethical approval. These are (1) Inconsistent definitions of research, (2) Perceptions of low-risk, (3) Perceived urgency due to emergency context, (4) Prior study or tool approval, and (5) Lack of knowledge about ethics review procedures.

**Conclusion:**

Institutional gaps in humanitarian governance structures are identified, urging the need for specialized ethics oversight mechanisms. The dynamic nature of humanitarian crises prompts nuanced approaches to ethical scrutiny, emphasizing policy initiatives to harmonize research and humanitarian governance frameworks and learning lessons from research ethics oversight in public health emergencies.

**Supplementary Information:**

The online version contains supplementary material available at 10.1186/s12910-025-01194-5.

## Introduction

Darfur, a region located in Western Sudan, has been marred by an armed conflict since 2003. As a result of that conflict, it is estimated that over 300,000 people have died, over 2.7 million people have been displaced from their homes and around 4.3 million people needed humanitarian assistance in Darfur [1, 2]. Moreover, 2.5 million children under the age of five have experienced food insecurity in Darfur, and an estimated 2.3 million Darfuri people being internally displaced within Sudan [3], and hundreds of thousands had become refugees in Chad, Egypt, Europe, and other countries [4–8].

International NGOs and UN agencies have been present in Darfur since the beginning of the conflict in 2003 to provide vital humanitarian aid, including food, shelter, and healthcare to the millions of people affected by the violence and displacement. However, the presence and actions of these organizations have not been without challenges. INGOs have faced significant challenges related to access to the most affected parts of the region, safety of staff, allocation of resources, and the fluctuating fund [9–12].

Sudan has witnessed notable developments that have influenced the humanitarian landscape in Darfur and beyond. One significant event is the political transition that took place in 2019, marked by the ousting of longtime President Omar al-Bashir and the establishment of a transitional government. This transition has brought renewed hope for stability and peace in the country, although challenges persist. However, this optimism was short-lived as Sudan experienced a fresh surge of conflict in April 2023. By October of the same year, the crisis had resulted in nearly 6 million internal displacements and over 1.4 million seeking refuge in neighbouring countries, exacerbating an already dire humanitarian situation. The region, alongside its neighbours, grappled with the challenge of accommodating large refugee and internally displaced populations. Internally, ongoing conflicts aggravated shortages of fundamental resources like food, water, and fuel, while healthcare services faced immense strain, leading to critical deficiencies in medical supplies.

Research and studies play a critical role in humanitarian settings like Darfur. They help to identify the specific needs of the affected population, including their basic needs such as food, water, and shelter, as well as their healthcare needs. Studies that examine the impact of humanitarian aid programs can help improve the effectiveness of relief efforts. This can include evaluating the distribution and utilization of aid, identifying gaps in service provision, and determining what changes are necessary to improve aid delivery. Such studies can also help identify promising approaches and practices that can be replicated across humanitarian contexts, and to providing decision-makers with the best available evidence [13].

In a previous article [14], we reported on the findings of a systematic review that was conducted “to assess the proportion of publicly available online reports of the research activities undertaken on humans in Darfur between 2004 and 2012 that mention obtaining ethical approval and/or informed consent” [14]. The results showed that out of the 68 eligible studies, as little as 9 (13.2%) reported gaining ethical approval and 29 (42.6%) that informed consent was obtained from the participants. While None of the 138 eligible reports in the Centre for Research on the Epidemiology of Disasters (CRED) hand search mentioned gaining ethical approval and 17 (12.3%) mentioned obtaining informed consent from their participants.

Based on reports included in the systemic review, we have concluded the review with five possibilities to explain these low percentages of reporting of ethical approval, which are: (1) These studies were exempted from ethics review, (2) Mentioning ethics review was not required, (3) Ethics review was considered by the researchers as if granted, (4) Pre-approved proposals were used, (5) The mention of the ethics review was not part of the template used.

This article delves into the reasons why not all studies conducted during Darfur’s armed conflict obtained ethics approval. Our qualitative study was conducted with key stakeholders involved in the planning, coordination, or review of research and humanitarian activities in Sudan between 2004 and 2012. It aimed to uncover the factors contributing to the lack of ethics approval for some studies during the Darfur conflict. It is crucial to highlight that the studies included in our analysis were those conducted within this specific timeframe, while the qualitative study was completed in 2018. The delay in the publication of our findings was influenced by several factors as detailed in the limitations.

## Methods

### Study design

A qualitative study design using focus groups and in-depth key-informant interviews was employed to investigate the reasons behind the low reporting of ethical approval in studies conducted in Darfur during the armed conflict.

### Sampling and participants’ recruitment

Purposive sampling was employed to ensure the inclusion of key stakeholders involved in the planning, coordination, or review of research and humanitarian activities in Sudan between 2004 and 2012. This approach targeted representatives from various institutions, including International Non-Governmental Organizations (INGOs), the Humanitarian Aid Commission (HAC), the Department of Research at the Federal Ministry of Health (FMOH) in Sudan, and the National Research Ethics Committee (NREC). Participants were reached out to by phone or visited in their offices to invite their participation in the study.

In this article, humanitarian agencies are defined as organizations that offer aid and assistance to people who are in need due to natural disasters, conflicts, or other crises. These agencies typically focus on providing emergency relief, such as food, shelter, medical care, and other essential services, to alleviate the suffering of affected populations. Examples of humanitarian agencies include the United Nations agencies, international non-governmental organizations, as well as non-governmental organizations involved in humanitarian efforts.

It’s important to note that participants were selected based on their institutional roles rather than direct involvement as researchers, planners, or reviewers of studies conducted during the study period. This decision aimed to ensure that participants possessed comprehensive knowledge about the research processes in Darfur. Imposing a condition of direct involvement would have limited the pool of eligible participants without significantly enhancing the study’s insights. Ethical considerations within institutions typically set the technical and ethical standards for surveys conducted in the field, which remain consistent across studies due to similarities in methods and tools.

The selection of INGOs was based on their research activities conducted in Darfur during the study period [14]. This approach ensured that participants represented organizations actively engaged in research endeavours relevant to our qualitative study’s objectives.

### Data collection

Semi-structured in-depth interviews and focus group discussions (FGDs) were carried out in Khartoum at the interviewees’ offices and the Federal Ministry of Health, respectively. The interviews and FGDs were conducted in both English and Arabic languages. The interview and focus groups’ topic guides were developed for this study [Supplementary file 1]. The interviews and FGDs were conducted by the first author, who is fluent in both English and Arabic languages. Therefore, there was no need for translation during the data collection process. The first author also transcribed the interviews and FGDs personally, ensuring accuracy and consistency in the transcription process. Transcription and translation were done sequentially, with the first author transcribing the data in the language it was conducted in and then translating it into the other language, if necessary. To ensure the accuracy of the transcriptions and translations, a rigorous quality control process was implemented, including cross-checking by a second author who is fluent in both languages and verification of key sections by participants themselves whenever possible. Any discrepancies or errors identified during this process were promptly addressed and corrected to maintain the integrity of the data.

In total, there were 38 participants: 5 interviewees (2 males and 3 females), and 33 participants in the FGDs (19 males and 14 females) (Table 1).

**Table 1:** Numbers of responses received from the invited participants in each respective category (*N* = 38)

Category of participants	No. of invitees	Invited for	No. of participants			The label used in the transcription
			Tot.	M	F	
1. The directors/heads of Sudan’s offices of relevant UN Specialised agencies and the Red Cross/Crescent	6	Interviews	1	1	0	UN
	5	FGDs	2	1	1	FGD-NGOs
3. The directors/heads of missions of selected INGOs	6	Interviews	1	1	0	INGO
	10	FGDs	8	4	4	FGD-NGOs
5. The directors of NGOs	20	FGDs	19	13	6	FGD-NGOs
6. Humanitarian governance bodies	3	Interviews	1	0	1	HGB
7. Research governance bodies			2	1	1	RGB
8. Relevant FMOH departments	6	FGDs	4	1	3	FGD-NGOs
Total	56		38	22	16	

FMOH: Federal Ministry of Health (Sudan); FGDs: Focus Group Discussions; HGB: Humanitarian Governance Body, which includes the Humanitarian Aid Commission (HAC); INGO: International Non-Governmental Organization; NGOs: Non-Governmental Organizations; NGOs: Non-Governmental Organization; RGB: Research Governance Body, which include the National Research Ethics Committee and the Research department at FMOH; UN: the United Nations

### Data analysis

We used inductive qualitative content analysis [15, 16]. This involved multiple readings of the transcripts to identify and categorise emerging themes relevant to the study’s central research question: “Why did not all studies conducted in Darfur between 2004 and 2012 obtain ethics approval?”

Our analytical approach began with systematically mapping the data to the main participant categories: governmental bodies including the HAC, the FMOH-RD, and the NREC, as well as NGOs’ and INGOs’ representatives. This focused categorisation enabled a more targeted analysis of the perspectives and experiences across these distinct groups.

We then undertook a rigorous coding process, followed by iterative re-coding, to identify major themes and patterns within the data. These emerging themes were systematically mapped back to the research question and cross-referenced with each other to explore similarities and differences across participant categories.

Through this inductive approach to interpretation, we aimed to develop and refine concepts and constructs directly from the data, offering a deeper understanding of the broader contextual meaning of our findings [17].

### Data validation

To address potential biases in data interpretation [18], we implemented several rigorous measures to bolster the trustworthiness and credibility of our findings. Firstly, we engaged a diverse group of participants with varying experiences, ensuring a nearly equal representation of both genders in our study [19, 20].

Throughout the research process, we incorporated key components of the research cycle specific to Darfur. These included ongoing member checking, where we verified the accuracy of our interpretations and the resulting themes and explanations with some of the participants via online meetings and exchange of emails. This iterative process allowed us to refine our understanding and ensure alignment with participants’ perspectives and ensured that our findings resonated authentically with the experiences and perspectives of our participants.

### Ethical considerations

This study strictly adhered to the ethical guidelines set forth by the Declaration of Helsinki [21] and the National Research Ethics Guidelines of Sudan [22]. Ethical approvals were granted by both the University of Birmingham’s research ethics committee and the National Research Ethics Committee (NREC) in Sudan.

Verbal informed consent was procured from all participants prior to their participation in interviews or focus groups. Participants were explicitly informed of the voluntary nature of their involvement and were assured of their right to withdraw from the study at any point before the analysis of their interview transcripts or FGDs, with the option to have their data removed.

To further ensure participant anonymity, rigorous measures were taken during data analysis. For instance, references to participants as representatives of the research governance bodies (RGB) were intentionally generalised to encompass the Research Directorate at the federal ministry of health, state ministries of health, and the national research ethics committee (NREC), without specifying individual positions. Similarly, references to representatives of International Non-Governmental Organizations (INGOs) were kept broad, considering the presence of over 100 international NGOs operating in Sudan, each with a large staff complement.

Given the dynamic nature of personnel in humanitarian settings and the possibility of participants having left their positions or even the country, we’ve made concerted efforts to ensure identities remain obscured.

## Results

The results are organized around five main themes that emerged from the data analysis (Table 2). Firstly, we discuss the inconsistent definitions of research observed among participants, highlighting the variations in how research activities were conceptualized and labelled. Next, we delve into perceptions of low risk associated with humanitarian studies, exploring the differing perspectives on the level of risk inherent in such activities. We then examine the perceived urgency due to the emergency context in Darfur, shedding light on the factors influencing the prioritization of rapid response over formal ethical review procedures. Additionally, we address the issue of prior study or tool approval, uncovering the practices surrounding the approval process for research conducted in humanitarian settings. Finally, we explore the lack of knowledge about ethics review procedures among participants, elucidating the challenges and barriers faced in navigating ethical considerations in research. Through these main themes, we aim to provide a comprehensive understanding of the complexities surrounding the decision-making process regarding the submission of humanitarian studies for formal ethics.

**Table 2:** Reasons for not submitting the humanitarian studies in Darfur for formal ethical approval

Main themes	
1. Inconsistent definitions of research 2. Perceptions of low-risk 3. Perceived urgency due to emergency context 4. Prior study or tool approval 5. Lack of knowledge about ethics review procedures	

### Inconsistent definitions of research

The understanding of what constitutes research activities varied significantly among participants, particularly evident among individuals affiliated with humanitarian agencies. Some participants hesitated to categorize their activities as “research,” opting instead to use alternative terms to describe their data collection activities. This hesitancy was notably expressed by individuals from humanitarian agencies, who emphasized the urgent nature of their work and its focus on providing rapid responses to emergencies.

INGO-1 stated, “In our organization, there is such research but not in Sudan’s mission. In other projects, there are studies, following these ethical guidelines and so and so. But for the rapid initial assessment, it is more related to providing a rapid response– emergency.” When questioned by the researcher about whether they considered their activities as research, INGO-1 responded, “No. Not research.”

However, this reluctance to label their activities as research was countered by representatives from research governance bodies, who advocated for a more stringent adherence to established definitions. These individuals emphasized the importance of aligning with recognized definitions of research, as outlined by organizations such as the World Health Organization, which encompass a broad spectrum of research types involving human subject involvement [23, 24].

*“[T]he reference definition for research or health research is the same as the definition in the World Health Organisation*,* which includes any of the types of [research]*,* whether epidemiological*,* and socio-behavioural*,* basic*,* and clinical*,* and socioeconomic… what matters* [is] *that all of them have human subject involvement” (RGB-1)*.

### Perceptions of low-risk

A significant contrast emerged in the perception of risk associated with humanitarian studies. While individuals from governmental governance bodies tended to view all studies as research inherently carrying some level of risk, individuals from non-governmental organizations (NGOs) held varying perspectives on the risk levels of such studies. Many NGO representatives considered humanitarian studies to be generally low-risk activities, particularly when they did not foresee direct harm and did not involve biological samples. This perspective stemmed from a broader belief among NGOs that in certain circumstances where potential harm was minimal, the ethical review process could be waived. However, this viewpoint sharply contrasted with the cautious approach of governmental governance bodies, which typically adopted a more conservative stance in evaluating the risk levels of humanitarian studies.

*“[…] where there is no harm and [researchers] do not take biological samples. This can be [an] exception from ethical [review]” (FGD-NGOs2/1).*

### Perceived urgency due to emergency context

The urgency of the humanitarian context in Darfur presented a notable divergence in perspectives regarding the application of ethical approval. NGOs emphasized the critical nature of their work, often employing terms like “emergency” and “rapid response” to underscore the need for immediate action. They expressed concerns that the lengthy procedures associated with ethical review could impede their ability to provide timely assistance. This urgency was encapsulated by statements such as “Any [displacement] camp for us means emergency [operation],” highlighting the imperative nature of their operations. Furthermore, NGOs voiced frustrations over bureaucratic delays for ethical review applications.

*“In Sudan*,* we have ‘tortoised’ (i.e.*,* very slow) procedures realistically. The period of two months to get the ratification to work in the Darfur region is long to the extent that what I want to study may have changed” (FGD-NGOs1/7).*

In contrast, representatives of governance bodies contested this urgency, asserting that the situation in Darfur no longer warranted emergency measures. They argued that ethics review procedures would not disrupt humanitarian aid efforts and emphasized the importance of ensuring ethical standards, even amidst pressing circumstances. Additionally, other UN agency representatives emphasized the necessity of upholding ethical considerations, asserting that the time required for ethics review could run parallel to technical and logistical preparations. They cautioned against sacrificing ethical standards in the name of urgency, emphasizing the importance of obtaining consent and adhering to ethical guidelines. This discourse highlighted the complex interplay between humanitarian urgency and ethical considerations in the context of Darfur.

*“The survey does not take place unless the situation has become a bit stable*,* and no people are dying… Ethics review will not disrupt humanitarian aid at all and will not lead that we lose lives” (HGB-1).*

*“Ethical [review] committee*^1^*cannot take a long time; not like the technical work. I think this (ethics review) can go parallel with that. Usually*,* I don’t agree with collecting this information ‘…’ in a hurry at the expense of having people’s consent or having these ethical considerations because unless you have this consent*,* I think all that you have done is unethical. […] I don’t agree with this [urgency] argument” (UN-1).*

### Prior study or tool approval

Several NGO representatives highlighted the close collaboration between their organizations and partner ministries as a form of implicit approval that they believed could substitute formal ethical review by NREC. Their argument rested on three key points. Firstly, they pointed out that governmental bodies routinely reviewed the data collection tools used by the organizations. If these tools were approved by these governmental bodies, then they questioned the necessity of further scrutiny by another governmental body, such as the NREC. Secondly, they emphasized that many governmental departments, including some within the Federal Ministry of Health (FMOH) where the Research Governance Bodies (RGBs) were situated, worked closely with the organizations in the field. They argued that if these departments, essentially neighbours to the RGBs, were not familiar with or adherent to research governance procedures, it would be unreasonable to expect the organizations to be fully informed or compliant. Lastly, they posited that the ethical considerations had already been adequately addressed and safeguarded through the ongoing monitoring and oversight provided by these governmental bodies.

*“All the parties go together: the [Humanitarian Aid] Commission*,* ‘UN agencies’*,* the concerned ministries*,* the governmental counterparts*,* they form Joint Assessment Missions. When ‘these are’ established*,* this means the tacit approval of the governmental agencies and means the tacit consent of the people who we want to assess. There is approval from all the sides to help these people” (FGD-NGOs1/4).*

Many participants indicated that the data collection tools (e.g. questionnaires) are standardised, and to them, this means they have already been reviewed and approved elsewhere.

*“It (the survey) used standardised [data collection] tool*,* already considered these ethical issues and it is used […] by other NGO’s missions*,* so there is […] no ethical issue raised during our assessments” (INGO-1).*

Representatives of some NGOs and INGOs argued that the absence of formal ethics review does not mean the absence of ethical standards. They gave examples of their procedures and organisational values and emphasised that the absence of ethics review did not render their work unethical.

*“We try to follow the ethical standards even without knowing that there is something called the Committee on Ethical Standards (sic) - [we have] Self-controls” (FGD-NGOs3/3).*

### Lack of knowledge about ethics review procedures

Participants across the board identified a lack of knowledge about the existence of the National Research Ethics Committee (NREC) and the national research ethics guidelines as a contributing factor to the failure to submit their work for ethics review. This lack of awareness was acknowledged by representatives from both the Research Governance Bodies (RGBs) and non-governmental organizations (NGOs). RGB representatives recognized this as a potential shortcoming, attributing it to a failure in advocacy efforts. They expressed a sentiment of missed opportunities for informing and encouraging stakeholders about the importance of ethical review procedures.

*“It is possible […] that they are genuinely not knowing (about NREC) it is possibly [one of our] shortcomings. We did not advocate. More advocacy and capacity building ‘are’ needed” (RGB-1).*

In contrast, RGB representatives suggested that NGOs may have purposefully avoided seeking ethical approval, reflecting a reluctance or unwillingness to engage in the process. This perspective challenges the notion that delays in the approval process were unjustifiable, asserting that NGOs cannot lament delays in a process they have not actively participated in. The sentiment conveyed is one of scepticism regarding NGOs’ intentions, implying that they may be using delays in the approval process as a convenient excuse, despite not having initiated the ethical clearance process. This viewpoint suggests a systemic issue of avoidance or neglect of ethical review procedures within certain sectors, raising questions about accountability and commitment to ethical standards.

*“If they (the NGOs) have the intention to submit it (the study)*,* they would ‘have submitted’ it and then the ‘ethical committees’ look into it*,* even if they fail to pass it because of time; they may have an excuse*,* but the problem is that it is a vindication*,* which they are putting for a procedure which they didn’t initiate. […] They could have asked that “Let’s have an ethical clearance” and then wait for it if it’s too long; then they will have the excuse that “we’ve asked for it*,* [but we got] no reply*,* [so] we started.” But they don’t*,* and I believe that they do not even consider doing that.” (RGB-2).*

### Limitations

While this study represents a pioneering effort to explore the complexities of ethical oversight in humanitarian research, it is essential to acknowledge several limitations that warrant careful consideration.

Firstly, the study’s scope and methodology are constrained by a temporal gap. The timeframe of the study (2004–2012) may not fully capture the current perspectives of stakeholders due to turnover in personnel within humanitarian settings. Additionally, the governance landscape governing humanitarian research may have evolved since the study period, potentially affecting the relevance of current stakeholders’ opinions compared to those involved in research activities in Darfur.

Secondly, the findings of this study are context-specific, and primarily relevant to Darfur during the conflict. While the insights derived from this study offer valuable understanding within this specific context, caution is advised when generalising these findings to other humanitarian settings. The unique geopolitical, cultural, and historical factors influencing research governance in Darfur may limit the generalizability of the study’s findings beyond this specific setting.

Furthermore, the representation from International Non-Governmental Organizations (INGOs) was lower compared to that of national NGOs and governmental bodies. This imbalance in representation may be attributed to the sensitivity of the topic to INGOs, particularly in the historical context where the former ruling regime expelled 13 INGOs and UN representatives from the country [25, 26] in response to publishing unfavourable findings about the humanitarian situation in Darfur. This imbalance could have potentially impacted the comprehensiveness of perspectives gathered in the study.

Lastly, methodological limitations should also be acknowledged. There may have been instances of social desirability bias, where participants might have been reluctant to disclose their lack of ethics approval or other potential shortcomings. Additionally, there might have been selection bias, with participants who agreed to participate potentially holding different perspectives than those who declined.

Moreover, some Focus Group Discussions (FGDs) may have been influenced by bias, with participants possibly hesitant to express views that could portray them or their organizations negatively. The size of the FGDs could also have influenced the dynamics and depth of discussions, potentially affecting the comprehensiveness of perspectives shared during these sessions.

On a final note, there was a lag between the end of the study in 2018 and its publication due to logistical considerations, such as prioritization of dissemination formats, timing considerations, and securing funding for publication.

## Discussion

Ethical considerations play an important role in guiding research conduct and ensuring the protection of participants’ rights and well-being. Within the dynamic landscape of humanitarian research, where urgency often intersects with complexity, navigating ethical challenges poses unique dilemmas.

Researchers often conduct studies as part of broader humanitarian efforts, regulated by distinct procedures that humanitarian governance and funding mechanisms follow to ensure the ethical conduct of activities, negating the need for formal ethics reviews. While it is universally acknowledged that humanitarian organizations adhere to specific principles, it is essential to recognize that these principles can vary significantly across different organizations. Some examples include the how principles like humanity, impartiality, and neutrality are embedded in humanitarian guidelines such as the Humanitarian Principles and the Fundamental Principles of the Red Cross and Red Crescent [27–29]. Similar principles are outlined by other international agencies and guideline like UNHCR humanitarian principles [30]and the SPHERE standards for humanitarian response [31]. This diversity arises due to variations in organizational mandates, cultural contexts, and operational approaches. However, a closer examination reveals gaps both in content and structure. The existing ‘Code of Conduct’ focuses on general humanitarian interventions, lacking specific guidance on research-related ethical issues. For example, the ethical issues related to ensuring the rights to respect autonomy, confidentiality, privacy and so on cannot be easily inferred and hence guided by such general principles. Furthermore, humanitarian clusters and meetings lack representation from bodies knowledgeable in research ethics, challenging the idea of replacing conventional research governance with existing humanitarian structures.

In this discussion, we explore the nature of ethical decision-making in humanitarian settings, drawing insights from our analysis of data gathered from stakeholders involved in research activities in Darfur.

Five main themes emerged from the data: inconsistent definitions of research, perceptions of low risk, perceived urgency due to the emergency context, prior study or tool approval, and lack of knowledge about ethics review procedures. Through these thematic lenses, we aim to discuss the complexities surrounding ethical decision-making processes and shed light on the factors influencing stakeholders’ attitudes and behaviours towards formal ethics review procedures.

By unpacking these themes and synthesizing key insights, we seek to contribute to ongoing discussions surrounding ethical governance in humanitarian research. Our analysis not only highlights the challenges and tensions inherent in balancing ethical imperatives with operational exigencies but also underscores the importance of fostering a culture of ethical awareness and accountability within the humanitarian research community. Through collaborative efforts and proactive engagement, we aim to chart a path towards more robust and ethically sound research practices in humanitarian contexts.

### Inconsistent definitions of research

The uncertainty surrounding the definition of research within humanitarian contexts reflects a fundamental ambiguity that permeates the field. While some participants, particularly those embedded within humanitarian organizations, expressed hesitancy in categorizing their activities as “research,” others from research governance bodies advocated for a more stringent application of established definitions. This discrepancy underscores the broader challenge of defining research within the complex and dynamic landscape of humanitarian interventions.

This ambiguity is not uncommon, as the literature indicates challenges in defining research during public health emergencies [32, 33]. Despite efforts to distinguish between ‘research’ and similar public health practices [34–36], defining the boundary remains elusive. In exploring the delineation between research and operational activities in acute humanitarian settings, it is important to consider the complexities involved.

One key consideration is the distinction between research and operational activities, which often blur in acute humanitarian settings. Rapid needs assessments and Joint Assessment Missions, for instance, primarily serve operational purposes, aiming to identify immediate needs and facilitate swift responses rather than generating generalizable knowledge. However, the absence of clear delineation criteria can lead to confusion and inconsistency in classification. Clarifying this boundary is essential to ensure ethical conduct and appropriate oversight while preserving the flexibility necessary for an effective humanitarian response.

### Perceptions of low-risk

The divergence in risk perception among participants underscores the subjective nature of risk assessment, particularly within the realm of humanitarian studies. While individuals from governmental governance bodies tended to adopt a cautious stance, viewing all studies as inherently carrying some level of risk, representatives from NGOs often took a contrasting view, regarding humanitarian studies as low-risk activities. This disparity in perspectives highlights the complexity of evaluating risk within the context of humanitarian research, where varying priorities and considerations come into play.

Moreover, participants emphasized the minimal risk posed by humanitarian research activities, pointing to the absence of adverse events in their studies as evidence. However, it is essential to recognize that the absence of documented adverse events does not necessarily imply the absence of risk. This observation underscores the importance of unbiased risk assessment by independent reviewers who possess adequate knowledge of the context in which the study will be conducted. By engaging impartial reviewers who are not directly involved in the study, researchers can mitigate potential biases and ensure a more thorough evaluation of risk factors, thereby enhancing the integrity and rigour of the ethical review process.

This discrepancy raises questions about the adequacy of current risk assessment mechanisms and the need for more objective criteria to guide ethical decision-making. Importantly, the perception of low risk should not be equated with the absence of risk. Even in seemingly benign studies, ethical considerations must be carefully weighed to safeguard the rights and well-being of participants. Objective risk assessment mechanisms, informed by contextual factors and expert input, can help mitigate biases and ensure the ethical conduct of research activities. Furthermore, ongoing monitoring and evaluation are essential to detect and address potential risks as studies unfold.

### Perceived urgency due to emergency context

The urgency of the humanitarian context in Darfur emerged as a significant factor shaping participants’ attitudes towards ethics review procedures. NGOs often cited time constraints and the imperative nature of their work as justifications for bypassing formal reviews. However, representatives from governance bodies contested this urgency, emphasizing the importance of upholding ethical standards even amidst pressing circumstances.

The argument for bypassing formal ethics reviews based on the humanitarian context and time-related factors prioritizes immediate humanitarian assistance over procedural requirements. However, governance body representatives highlight two main factors that challenge this perspective: the longevity of the Darfur crisis and the relative stability of affected populations. They argued that the methods used in humanitarian surveys, such as multistage cluster sampling, indicate the presence of relatively stable demographic settlements within identifiable geographical units [37, 38]. While humanitarian needs can intensify due to events like hostilities, such urgencies are episodic rather than continuous, especially in longstanding armed conflict like that in Darfur. Moreover, they argue that formal ethics review processes are less resource-intensive compared to other logistical preparations for such surveys.

While this perspective underscores the practical challenges of integrating formal ethics reviews into humanitarian research, it also raises questions about the urgency of all humanitarian studies. In instances such as the displacement of large numbers in a brief time or disease outbreaks necessitate immediate action [6], waiting for the standard two-month period to obtain ethical approval could have devastating consequences, leading to increased morbidity and mortality rates [39, 40].

While the urgency of humanitarian response is undeniable, ethical considerations remain critical. Flexible ethical frameworks and review models can help strike a balance between urgency and ethical rigour, ensuring that research activities adhere to established ethical principles. This could include the establishment of expedited review procedures for urgent humanitarian research, or the pre-approval of standard methodologies commonly used in emergency assessments, like those suggested in other public health emergencies [41, 42]. Moreover, stakeholders must engage in proactive dialogue to develop context-sensitive guidelines that address the unique challenges posed by humanitarian emergencies without compromising research integrity.

### Prior study or tool approval

The reliance on implicit approval mechanisms, such as collaboration with partner ministries, raises questions about the adequacy of existing ethical safeguards within humanitarian organizations. While some argue that existing governance and funding mechanisms inherently ensure ethical conduct, others contend that formal oversight mechanisms are necessary to uphold research integrity.

The absence of specific guidance on research-related ethical issues within humanitarian frameworks underscores the need for comprehensive ethical guidelines tailored to the unique challenges of the field. Collaborative efforts between humanitarian organizations, research governance bodies, and other stakeholders are essential to develop and implement robust ethical frameworks that ensure the rights and well-being of participants are upheld.

### Lack of knowledge about ethics review procedures

The pervasive lack of knowledge about ethics review procedures, particularly regarding the existence of the National Research Ethics Committee (NREC), poses a significant barrier to ethical research conduct. While some attribute this gap to a failure in advocacy efforts, others suggest a systemic issue of avoidance or neglect of ethical review procedures within certain sectors.

Addressing this lack of knowledge requires concerted efforts to improve education and awareness about ethical review processes. Stakeholders must engage in capacity-building initiatives and promote a culture of ethical research conduct across all sectors. Furthermore, transparency and accountability mechanisms are essential to ensure that ethical standards are upheld and that researchers are held accountable for their actions.

### Recommendations

Based on our analysis of ethical decision-making in humanitarian research in Darfur, we offer the following consolidated recommendations to bolster ethical oversight of research during armed conflicts, such as the one witnessed in Darfur between 2004 and 2012 to enhance ethical governance and ensure the protection of participants’ rights and well-being:

#### Harmonized integrated regulatory oversight

The international humanitarian community should collaborate with governments in conflict-affected regions to establish and enforce regulatory frameworks governing research in humanitarian settings. This includes developing laws, policies, and guidelines that outline ethical standards and procedural requirements. Governments should support regulatory bodies to enhance their capacity for effective oversight and response to ethical challenges. Proactive regulatory oversight can promote ethical research conduct while fostering innovation in humanitarian settings. Develop clear guidelines for emergency research ethics through empirical evidence and local community involvement. These guidelines should define research in emergency settings, include risk assessment criteria, and address ethical considerations specific to conflict zones and reflective of the local communities’ moral values and priorities. Foster collaboration between humanitarian agencies, research institutions, and local stakeholders to embed ethical considerations in early-stage research planning and throughout its implementation in emergency contexts.

This includes working collaboratively to agree on common oversight models like pre-approval of research tools and protocols to encourage the pre-approval of commonly used research tools and protocols for emergency settings. This pre-approval can be conditional, subject to rapid modification in response to significant contextual changes [43, 44]. Alongside, we recommend a harmonized approach to research and humanitarian governance to promote the adoption of a unified approach aligning research governance with humanitarian endeavours. This approach should navigate the complexities of research in conflict-affected areas while upholding ethical standards.

#### Strengthening ethical governance within humanitarian organisations

Humanitarian organizations should engage with governments, agencies, and local communities to strengthen ethical governance and regulatory oversight of research activities. This includes providing technical expertise, capacity-building initiatives, and financial support to enhance regulatory frameworks. Advocate for the adoption of ethical guidelines and best practices in humanitarian research to promote transparency, accountability, and respect for participants’ rights and well-being. Collaborate with diverse stakeholders to create an enabling environment for ethical research conduct in humanitarian settings.

Ethics training for humanitarian workers is essential by integrating ethics training into standard preparation programs for humanitarian workers and researchers operating in conflict zones. This training should encompass ethical research principles, the significance of ethics approval, and the requisite procedures.

#### Support context-sensitive approaches

Develop context-sensitive guidelines and oversight mechanisms that address challenges in humanitarian emergencies while upholding ethical principles. Establish interim ethical oversight mechanisms in the absence of functioning governments, ensuring ethical research conduct in conflict-affected areas. Collaborate with international organizations and humanitarian agencies to uphold ethical standards. Establish specialized ethics committees for conflict zones with expertise in conflict and emergency settings. Implement a fast-track process for ethical review to balance swift response with participants’ rights and welfare.

These committees should use a streamlined ethics approval process that implement a fast-track process for the ethical review of research during emergencies that balances the imperative for swift response with the need to uphold participants’ rights and welfare. These committees should possess specialized expertise to address the unique challenges of research in such environments and facilitate prompt impartial decision-making. Additionally, these regulatory frameworks should prioritize flexibility and efficiency, ensuring that the research approval process is streamlined and accessible. By implementing clear and user-friendly procedures, governments can facilitate timely approval of research proposals, allowing humanitarian research to proceed without unnecessary delays.

These recommendations seek to fortify the ethical framework governing research in armed conflict and emergency settings, safeguarding the rights of research participants and upholding the integrity of the research process amidst challenging circumstances.

## Conclusion

This research aimed to explore the ethical dimensions of humanitarian research in complex settings, focusing on the case study of Darfur. By examining stakeholders’ perspectives, the study sought to uncover key insights into ethical decision-making processes and governance challenges within the humanitarian research landscape.

The results revealed several significant themes, including inconsistent definitions of research, perceptions of low risk, perceived urgency due to emergency contexts, reliance on implicit approval mechanisms, and lack of awareness about ethics review procedures. Stakeholders expressed divergent views on these issues, highlighting the complexity of ethical decision-making in humanitarian settings.

The findings have important implications for both academic discourse and practical applications in the field of humanitarian research. They underscore the need for tailored ethical guidelines and regulatory frameworks that balance flexibility with rigor, particularly in resource-constrained and conflict-affected environments. Moreover, the insights gained can inform future research and contribute to the ongoing dialogue on ethical governance in humanitarian contexts.

Stakeholders must take proactive steps towards enhancing ethical governance in humanitarian research. This includes advocating for policy changes, strengthening regulatory oversight and research governance, and fostering a culture of ethical responsibility within research organizations and funding bodies. By working collaboratively, we can strengthen ethical practices and uphold the highest standards of research conduct in humanitarian contexts.

## Electronic supplementary material

Below is the link to the electronic supplementary material.

Supplementary Material 1

## Data Availability

Data is provided within the manuscript or supplementary information files.

## Footnotes

1 The participant was referring to the NREC.

## Abbreviations

FGD: Focus Group Discussion
FMOH: Federal Ministry of Health (Sudan)
HAC: Humanitarian Aid Commission
HGB: Humanitarian Governance Body
INGO: International Non-Governmental Organization
NGO: Non-Governmental Organization
NREC: National Research Ethics Committee
RGB: Research Governance Body
UN: The United Nations
