# Supplementary material for: Why did not all studies conducted during Darfur’s armed conflict obtain ethics approval? Insights from a qualitative study
Source: BMC Med Ethics. 2025 May 6;26:58. doi: 10.1186/s12910-025-01194-5 (PMC12054246; doi:10.1186/s12910-025-01194-5)
Supplement: Supplementary file 1 — Supplementary Material 1 [file 12910_2025_1194_MOESM1_ESM.docx]

## The topic guide for the INGOs interviews

1. Self-introduction (5 mins), the researcher will
   - Thank interviewee for taking part in the research.
   - Introduce himself, and suggest that the interview will last up to 40 minutes
   - Explain purpose of research and few ground rules (e.g. breaks if needed, mobile phones)
   - Reassure re: confidentiality
   - presentation of the information sheet, and gain written consent

Topic discussion (30 minutes)

1. Please tell me about the activities that your institution (organization) conducted in Darfur between 2004 and 2012?

Prompts:

Did any of these activities involve the collection of personal data and/or any kind of biological samples from the target population?

1. Can you tell me about how your institution prepared for the conduct of these activities?

PROMPTS: what kind of committees or departments were responsible for these preparations? Please tell me more about their structure and hierarchy. (Probe: do they follow you directly or under other department)

1. What did you or your sponsors consider were the essential requirements to be fulfilled for any project to be conducted?

PROMPTS: are they technical? Logistic? Financial?

1. Please tell me about any changes that have taken place in the process of reviewing and approving the research undertaken by your institution since 2004, or since you have been in this post, if any?

Prompt: if no, go to Q6 directly. If yes, ask for details in the changes in terms of:

- What initiated such changes?
- What did these changes target? E.g. technical issues, methodological issues, or ethical issues
- How were these changes applied?
- How did they affect the actual undertaking of research in the field?

1. Thinking about your time in your current position, please tell me about anything that any of your data-collection teams have encountered that they (or yourself) considered to raise some ethical issues?

Prompt, if yes: can you give one or two examples of these issues

1. Do you think that there should be prior ethical review of activities that involve the collection of personal data or some samples like urine, stool, or blood? (If NO, go to Q 12)

Prompt: if yes, ask the following questions and if no go to Q 12

1. Have you had these activities ethically reviewed?

Prompt: refer to the examples from the findings of the systematic review with studies that were undertaken by the interviewee’s institution, if without ethical approval

1. How do have these activities ethically reviewed? By whom? Inside the NGO or outside it?
2. Do you face any difficulties in having these activities ethically reviewed?
3. If yes, what are the difficulties that you face in having these activities ethically reviewed?

Prompts: no committees? The national committee is not efficient? No local committees in Darfur? Time consuming? Lack of clear guidance?

1. If no, why do you think that these activities do not need to be ethically reviewed?

Prompts: they are not research? emergency situation and ethical review is time consuming? No committees? No guidelines? Not requested by the NGO sponsors?

1. If the answer is ‘not research’, then what would define the research that should be ethically reviewed?

Prompt: how do you think that these activities differ from the characterization of research that you have just described?

Are there any conditions that would make your NGO consider submitting this kind of activity to ethical review?

1. For other options, then say “if I got your point clear, you think that (fill with the answer provided to the question or prompt) is the main reason for not considering your NGO’s activities for ethical approval. Am I right?”

If yes, go to Q 15, if no, then say, “sorry for not getting your point clearly, so can you please restate to me the main reason why your NGO would not consider these type of activities to ethical review”

1. Then if the (option chosen in Q 13) is resolved, would you consider applying these activities to ethical review?
2. Prompt: if yes, ask: how would you suggest the best approach to ethical review of humanitarian activities that involve the collection of personal data and/or biological samples?
3. Prompt: If answer to Q 15 is No, ask: so what do you think need to be resolved to consider these activities for ethical review?

**Summary and closing (5 minutes)**

1. The PI will summarise conversation and what has been discussed throughout the interview, the ask the interviewee:

• Is there anything else that you’d like to add or discuss?

• Any questions?

Thanks and close

## The topic guide for the INGOs’ research officials’ interviews

1. Self-introduction (5 mins), the PI will
   - Thank interviewee for taking part in the research.
   - Introduce self and notify participant that the interview will last up to 40 minutes
   - Explain purpose of research and few ground rules (e.g. breaks if needed, mobile phones)
   - Reassure re: confidentiality
   - presentation of the information sheet, and have the consent signed

**Topic discussion (30 minutes)**

1. Please tell me about the structure and functions of your department/committee?

Prompt: date, staff/membership, functions, facilities, meetings, workload, etc.

Prompt: relation with the UN agencies and the INGOs

1. The Sudanese (national) research ethics guidelines state that “international research done in Sudan should be only reviewed and approved by the national committee”, do you think that this includes the research undertaken by the INGOs in Darfur?

Prompt: given that these activities involve the collection of personal data and sometimes biosamples from the Sudanese citizens (If No, go to Q 6)

1. If yes, then can you tell me how did your department/committee manage to ethically review these activities since 2004, or since you have had this post?

Prompt: refer to the findings of the systematic review about how many studies were ethically reviewed by which committees? Number of studies submitted for ethical review from the INGOs in Darfur for the last year, for example.

1. If no (to Q 3), why do you think that these activities that involve the collection of personal data and/or biological do not fall within the characteristics specified in the guidelines?
2. Please tell me know how your department is currently handling the protocols for research to be undertaken in Darfur?

PROMPT: compare to the answer of Q4.

1. On a scale of 1-5, where 1 is very unsatisfied and 5 as very satisfied, how would you rate the overall role of your department/committee in regard to the oversight and coordination of the research activities in Darfur?
2. Why did you give such a level of satisfaction?
3. Do you think that the current structure of the department needs improvement in relation to research conducted in Darfur? [if yes, go to Q12]
4. Do you think that the current coordination and ethics review procedures needs improvement in relation to research conducted in Darfur? [if yes, go to Q12]
5. Do you think that the current ethics guidelines need improvement in relation to research conducted in Darfur? [if yes, go to Q12]
6. **PROMPT:** if his rate in Q7 is 3 or less, OR if his answer on Q9, Q10, or Q11 is ‘Yes’.

Ask: What do you think needs to improved regarding [structure/procedures/guidelines] on the research undertaken in Darfur? **NOTE:** make sure all the three questions (Q9 on structure, Q10 on coordination, and Q11 on guidelines are asked)

**Summary and closing (5 minutes)**

1. The PI will summarise conversation and what has been discussed throughout the interview, the ask the interviewee:

• Is there anything else that you’d like to add or discuss?

• Any questions?

Thank and close

## The topic guide for the Focus Groups Discussions

There will be two approaches to the FGDs. The PI may shift between them as needed in order to make the participants come with as much interaction and information as possible. The use of two approaches to the FGDs aims at generating the needed amount or path of data relevant to the project. The first approach will be using a case scenario and have it read to them, and then focus on attitudes to the case scenario. The second approach is a stepwise approach that uses probing open ended questions in relation to their previous experiences as research participants.

1. Self-introduction (5 mins), the PI will
   - Thank interviewee for taking part in the research.
   - Introduce self and notice that the interview will last up to 45 minutes
   - Explain purpose of research and few ground rules (e.g. breaks if needed, mobile phones)
   - Reassure the need for confidentiality and to refrain from sharing any information shared within the discussion outside it
   - presentation of the information sheet, and have the consent forms signed

### Approach A: discussion of a scenario, or case study

**Topic discussion (30 minutes)**

1. The PI (or the research assistant in the female FGD) will be reading an example of an informed consent that was used in a household survey that was undertaken by an international humanitarian aid agency in Darfur between 2004 and 2012. The example informed consent will be that of the Sudan Household Health Survey that was conducted in Darfur in 2006, because it involved the collection of personal data as well as performing anthropometric measures on children under 5 years old in the selected households.

This is the excerpt of the informed consent used in the household questionnaire(Damian and Damundu 2007):

“Household questionnaire:

We are from the Sudan household health survey which is concerned with family health and education. I would like to talk to you about this. The interview will take about 45 minutes. All the information we obtain will remain strictly confidential and your answers will never be identified. During this time i would like to speak with the household head and all mothers or others who take care of children in the household.

May I start now? *If permission is given, begin the interview.*”

Examples of the probing questions to initiate discussion of this consent are:

1. What do you think about this informed consent form?
2. Tell me about your previous experiences with data collectors asking your permission to collect data from you
3. What else would like to be considered by the data collectors when they approach you in similar (research) activities
4. The PI will summarise conversation and what has been discussed throughout the interview, the ask the interviewee:

• Is there anything else that you’d like to add or discuss?

• Any questions?

Thank and close

### Approach B: previous personal experiences

**Topic discussion (30 minutes)**

1. Tell me about your experience with one of the times where the humanitarian workers came to your household and asked you questions about your health, food, education, etc.

Prompt: any biological samples taken? Blood? Urine? Stool?

1. Did they ask for permission before they start asking questions? (If No, go to 6)
2. If yes, Can you describe to me how did they take these permissions? Prompt: which details told? In which language? Who took the permission?
3. If no, what made you let them ask you questions or take samples?

Prompt: trust in the NGO? Needed to answer to get aids? the camp leader told them to? Other reasons? explore

1. Do you know what happened to the data or samples you gave?

Prompt: if yes, let them give examples of what could have happened to the data and the samples

1. If no, what do you think the data and samples could have been used?
2. Are there any data collection activities currently going in your camp?
   - If yes, how they are done?
   - Do you think there are any problems related to them? [Yes/No]

Prompt: if Yes, can you elaborate more on what do you see as problems?

1. If yes, you want them to continue as they are or what are your suggestions to make these activities better to you?
2. If no, why not?

Thanks and close
